# Meta-analysis of the influence of *TM6SF2* E167K variant on Plasma Concentration of Aminotransferases across different Populations and Diverse Liver Phenotypes

**DOI:** 10.1038/srep27718

**Published:** 2016-06-09

**Authors:** Silvia Sookoian, Carlos J. Pirola

**Affiliations:** 1 Department of Clinical and Molecular Hepatology, Institute of Medical Research A Lanari-IDIM, University of Buenos Aires- National Scientific and Technical Research Council (CONICET), Ciudad Autónoma de Buenos Aires, Argentina; 2 Department of Molecular Genetics and Biology of Complex Diseases, Institute of Medical Research A Lanari-IDIM, University of Buenos Aires-National Scientific and Technical Research Council (CONICET), Ciudad Autónoma de Buenos Aires, Argentina

## Abstract

A nonsynonymous E167K (rs58542926 C/T) variant in *TM6SF2* gene was recently associated with nonalcoholic fatty liver disease (NAFLD). We explored the association between E167K and plasma concentrations of alanine (ALT) and aspartate (AST) aminotransferases through a meta-analysis. We also estimated the strength of the effect across diverse liver phenotypes, including NAFLD and chronic viral hepatitis; fourteen studies were included. We found that ALT (*p* = 3.2 × 10^−6^, *n* = 94,414) and AST (*p* = 0007, *n* = 93,809) levels were significantly associated with rs58542926 in NAFLD. By contrast, rs58542926 was not associated with either ALT (*p* = 0.24, *n* = 4187) or AST (*p* = 0.17, *n* = 2678) levels in four studies on chronic hepatitis. In conclusion, the results of the pooled estimates in patients with NAFLD showed that carriers of the T allele (EK + KK), when compared with homozygous subjects for the C allele (EE genotype) have increased levels of aminotransferases; however, this increase represents –2.5 (9.8%) and 1.2 (5%) IU/L of ALT and AST respectively, which is fairly small compared with the large effect of *PNPLA3-* rs738409-G allele that is associated with a –28% increase in serum ALT.

Plasma concentrations of alanine (ALT) and aspartate (AST) aminotransferases have been classically regarded as markers of liver injury, including a wide range of etiologies from viral hepatitis to nonalcoholic fatty liver (NAFLD)^1^. Indeed, under the assumptions that liver cell membrane damage is associated with the subsequent leakage of intracellular enzymes into the circulation, in addition to the fact that aminotransferases are highly expressed in hepatocytes, the measurement of circulating ALT and AST enzymatic activity is commonly used in clinical practice to evaluate and monitor the course of any acute or chronic liver disease associated with liver damage.

The large body of evidence derived from epidemiological studies on the prevalence of obesity, type 2 diabetes (T2D), and cardiovascular disease (CVD) has uncovered an unexpected but biologically plausible association between aminotransferases and all of the individual components of the metabolic syndrome (MetS), as recently reviewed^2^. The findings obtained by the Framingham Offspring Heart Study showed that ALT levels are not only highly correlated with incident MetS, but are also significantly associated with the increased risk of T2D and incident CVD over a 20-year follow-up period^3^. Recent results from a patient-oriented-research study from our group showed that in the context of abnormal hepatic triglyceride accumulation, circulating aminotransferases rise putatively as a consequence of the increased tissular reactions of transamination in order to cope with the liver metabolic derangement^4^. Hence, the ALT and AST levels might be regarded as sensors of global metabolic deregulation, including mitochondrial energetic control^4^.

Like all circulating substances, the plasma levels of aminotransferases are highly variable and are affected by a myriad of factors including genetic predisposition^5^,^6^; for instance, the estimated heritability of ALT is about 33%^7^. The first genome-wide association study (GWAS) of plasma liver-enzyme levels identified two loci influencing ALT levels, one of which included two imputed-nonsynonymous SNPs within patatin-like phospholipase domain containing 3 (*PNPLA3*) (rs738409 Ile148Met, *p* = 3.7 × 10^−10^ and rs2294918 Lys434Glu, *p* = 6.0 × 10^−4^)^8^. This finding was further replicated in a subsequent larger GWAS (n = 61,089 individuals) demonstrating a remarkably similar effect for the rs738409 (*p* value for the association of ALT 1.2 × 10^−45^)^9^. Likewise, the results of the first GWAS on NAFLD also found an association between the rs738409 and serum ALT levels, although this association was only restricted to the Hispanic group, which was the ethnic group with the greatest prevalence of hepatic steatosis in the recruited population^10^. Summarized evidence from the following-up candidate-gene association studies demonstrated that carriage of the homozygous state for the rs738409-G allele is associated with a ~28% increase in serum ALT levels^11^.

A recent exome-wide association study of liver fat content showed that rs58542926 (E167K), a nonsynonymous variant located in *TM6SF2* (Transmembrane 6 Superfamily Member 2), was also associated with modest *p* values for the association with ALT but not AST in the Dallas Heart Study and The Dallas Biobank^12^, while it also bore a large significance, at least for ALT (*p* = 7.6 × 10^−14^) in the Copenhagen Study^12^. A concomitant GWAS on genes influencing lipid traits also found the rs58542926 associated with total cholesterol levels and myocardial infarction risk^13^. Successive explorations of the association between E167K and NAFLD showed that this variant has a modest effect on liver fat accumulation, and also a dual and opposite role in protecting against CVD and conferring risk for NAFLD^14^.

Furthermore, conflicting and non-replicated results found in some^15^,^16^,^17^ but not all of the studies^18^ that the E167K variant could be associated with steatosis in patients with the hepatitis C virus (HCV) as well; nevertheless, the associations with liver enzymes could not be demonstrated in patients with chronic hepatitis.

Interestingly, the results from the two above-mentioned GWAS on liver enzymes^8^,^9^ do not appear to suggest any significant association between E167K or any other variant in the linkage disequilibrium in the *TM6SF2* locus- and aminotransferases. Hence, whether the rs58542926 has any effect on the circulating levels of ALT or AST is still unknown. As the literature shows conflicting and inconclusive results, our primary purpose was to explore the putative association between the rs58542926 variant and plasma levels of aminotransferases by a meta-analysis of existing data. In addition, we estimated the strength of the effect of rs58542926 on both circulating ALT and AST across different populations and diverse liver phenotypes, including NAFLD and chronic viral hepatitis.

## Results

We evaluated fourteen studies that were identified using the search strategy described in Supplementary Fig. 1. Characteristics of studies included in the meta-analysis of liver enzymes in NAFLD^12^,^16^,^19^,^20^,^21^,^22^,^23^,^24^,^25^,^26^,^27^ (*n* = 11) are shown in Table 1, while those included in the meta-analysis of patients with viral hepatitis^15^,^16^,^17^,^18^ (*n* = 4) are summarized in Table 2.

Basic details of the included studies, including location, main clinical descriptors and sample size, and also major concerns or putative bias of the studies are summarized in Tables 1 and 2. In addition, Tables 1 and 2 include information on quality and methodology of the included studies, specifically, putative selection bias of the study design and setting, which is required in the HuGENet guidelines. Selection criteria, reference test, blind assessment of the reference test, and the availability of clinical data were disclosed in the majority of the studies.

### Study Characteristics

Eleven studies included in the meta-analysis were hospital-based^15^,^16^,^17^,^18^,^19^,^20^,^21^,^22^,^23^,^24^,^27^, while the other three were population-based^12^,^25^,^26^ studies; three studies included pediatric population^20^,^21^,^25^.

Genotyping for rs58542926 was carried out using a Taqman assay^15^,^17^,^18^,^19^,^21^,^22^,^23^,^24^,^26^,^27^, Sequenom MassARRAY system and iPLEX Gold chemistry^16^, automated sequencing^20^, Exome BeadChip^25^, and specific Illumina arrays (Illumina, CA, USA)^12^.

Concordance with Hardy-Weinberg equilibrium (HWE) was observed in all the studies as stated by the authors; specific assessment of departure from HWE was further performed according to the genotype frequencies reported in any paper whenever this information was not disclosed.

### The influence of TM6SF2 E167K variant on plasma concentration of aminotransferases in subjects with NAFLD

Associations for the plasma levels of ALT were extracted from eleven studies^12^,^16^,^19^,^20^,^21^,^22^,^23^,^24^,^25^,^26^,^27^, while the associations for plasma levels of AST were extracted from nine studies^12^,^16^,^19^,^20^,^21^,^23^,^24^,^26^,^27^. The mean values of ALT and AST, according to the genotypes of the dominant model of rs58542926 in each study are disclosed in Table 1.

**Figure 1:**
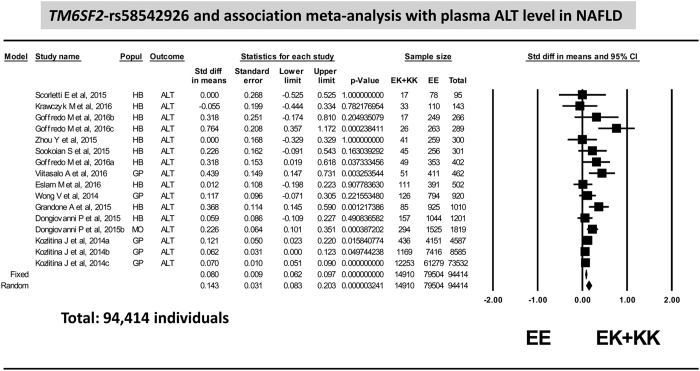
Association analysis on plasma level of ALT in patients with NAFLD: forest plot of rs58542926 variant (homozygous EE vs. EK + KK) and random and fixed effect models. The effect indicates the standardized mean difference, the standard error and the corresponding lower and upper limits, and sample size of each study according to the dominant model of inheritance. Studies were ordered by sample size. The first author of the study and the year of publication are shown after the study name; Dongiovanni, 2015 HB: stands for hospital-based study on NAFLD; Dongiovanni, 2015b: SOS Study (Swedish Obese Subjects); Kozlitina, 2014a: Dallas Heart Study; Kozlitina, 2014b: Dallas Biobank; Kozlitina, 2014c: Danish population Copenhagen Study; Goffredo 2015a: Caucasians, Goffredo 2015b: African Americans and Goffredo 2015c: Hispanics. Popul: indicates design features, GP: general population, HB: hospital-based, MO: morbid obese subjects. In the graph, the filled squares stand for the effect of individual studies, and filled diamonds express combined fixed and random effects.

We found that the plasma concentration of ALT was significantly associated with rs58542926 variant (random model *p* = 3.2 ×10^−6^) (Fig. 1) without evidence of publication bias (*p* = 0.44) in a sample of 94,414 individuals of both genders. The analysis revealed a significant heterogeneity (*p* = 0.0032, *I*^2^: 58.1) that by the sensitivity analysis it was primarily attributed to the studies including pediatric population^20^,^21^,^25^. Results on the ALT levels stratified by age are shown in Supplementary Fig. 2; heterogeneity disappeared when studies that included children were grouped apart in the analysis (*p* = 0.45, *I*^2^: 0).

**Figure 2:**
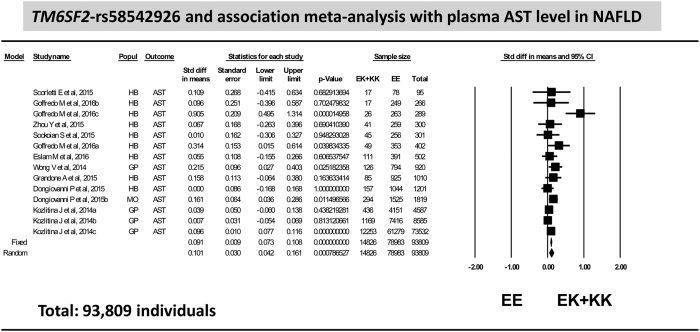
Association analysis on plasma level of AST in patients with NAFLD: forest plot of rs58542926 variant (homozygous EE vs. EK + KK) and random and fixed effect models. The effect indicates the standardized mean difference, the standard error and the corresponding lower and upper limits, and sample size of each study according to the dominant model of inheritance. Studies were ordered by sample size. The first author of the study and the year of publication are shown after the study name; Dongiovanni, 2015 HB: stands for hospital-based study on NAFLD; Dongiovanni, 2015b: SOS Study (Swedish Obese Subjects); Kozlitina, 2014a: Dallas Heart Study; Kozlitina, 2014b: Dallas Biobank; Kozlitina, 2014c: Danish population Copenhagen Study; Goffredo 2015a: Caucasians, Goffredo 2015b: African Americans and Goffredo 2015c: Hispanics. Popul: indicates design features, GP: general population, HB: hospital-based, MO: morbid obese subjects. In the graph, the filled squares stand for the effect of individual studies, and filled diamonds express combined fixed and random effects.

Likewise, circulating AST levels were significantly associated with rs58542926 variant (random model *p* = 0.00079, n = 93,809) (Fig. 2) without any evidence of publication bias (*p* = 0.33) but with evidence of heterogeneity (*p* = 0.004, *I*^2^: 57.3) that was also successfully solved when the studies that included pediatric population^20^,^21^ were grouped apart in the analysis (*p* = 0.12, *I*^2^: 36.9); the results on AST levels stratified by age are shown in Supplementary Fig. 3.

### The influence of TM6SF2 E167K variant on plasma concentration of aminotransferases in subjects with viral hepatitis

The analysis included a total of 4187 subjects from which the values of ALT according to E167K genotypes could be extracted^15^,^16^,^17^,^18^; this sample included 3680 patients with HCV and 507 patients with HBV. Only two studies reported data on AST levels^15^,^16^. The mean value of ALT and AST according to genotypes of the dominant model of rs58542926 in each study is disclosed in Table 2.

**Figure 3:**
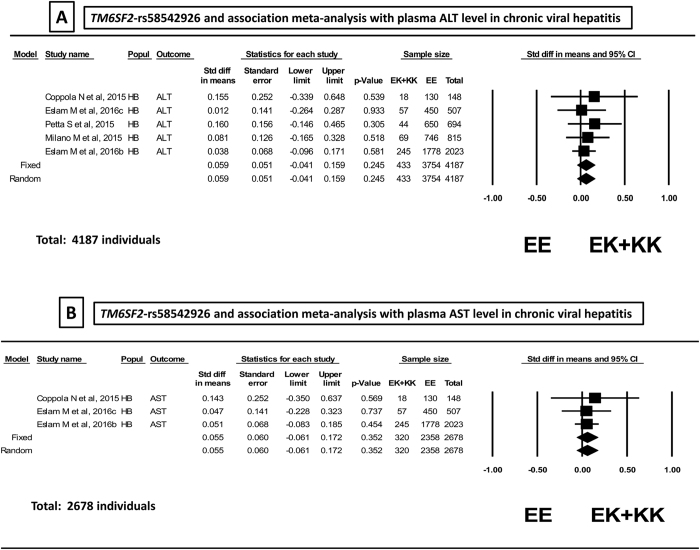
Association analysis on plasma level of ALT (panel A) and AST (panel B) in patients with chronic hepatitis (**B**,**C**): forest plot of rs58542926 variant (homozygous EE vs. EK + KK) and random and fixed effect models. The effect indicates the standardized mean difference, the standard error and the corresponding lower and upper limits, and sample size of each study according to the dominant model of inheritance. Studies were ordered by sample size. The first author of the study and the year of publication are shown after the study name. Eslam 2016a: NAFLD, Eslam 2016b: chronic hepatitis (**C**), Eslam 2016c: chronic hepatitis (**B**). HB: hospital-based. In the graph, the filled squares stand for the effect of individual studies, and filled diamonds express combined fixed and random effects.

Of note, the rs58542926 variant was not associated with ALT levels either in the fixed or random (*p* = 0.245) models (Fig. 3A) in the total sample of 4187 individuals without evidence of heterogeneity (*p* = 0.94, *I*^2^: 0) or publication bias (*p* = 0.22). Neither was the rs58542926 variant associated with plasma AST levels (fixed or random model *p* = 0.172) in a smaller sample that included 2678 individuals without evidence of heterogeneity (*p* = 0.94, *I*^2^: 0) or publication bias (*p* = 0.296) (Fig. 3B).

## Discussion

We explored the influence of rs58542926, a missense variant of *TM6SF2,* which is involved in the regulation of lipid metabolic process, on the concentration of aminotransferases in the circulating compartment. Interestingly, by means of a comprehensive and free from bias meta-analysis of the published evidence, we found that the rs58542926 variant exerts a moderate but statistically significant effect on the circulating levels of both ALT and AST in patients with NAFLD, but not in chronic viral hepatitis.

### Limitations and quality of the evidence

The results of this meta-analysis show no evidence of publication bias; assessment of completeness of information, validity of individual studies, and analytic value of the test investigated suggest that the overall quality and methodology of studies was high. Nevertheless, some potential limitations deserve to be discussed. First, the presence of heterogeneity may potentially restrict the interpretation of the pooled risk estimates, particularly concerning the association of the variant with ALT and AST in NAFLD. However, the random effect model that does not depend on heterogeneity yielded a significant result pertaining to the association with both enzymes. More importantly, after performing a sensitivity analysis in the complete dataset, we observed that the heterogeneity was explained by three studies that enrolled pediatric population^3^,^20^,^25^. One potential explanation could be given by the fact that a large proportion of children and adolescents included in the above-mentioned studies were eligible for inclusion if they were obese or had any metabolic perturbation, including insulin resistance. Thereby, selection bias could explain why studies that involved pediatric population have introduced heterogeneity into the main joined analysis. On the other hand, the fact that pediatric-NAFLD differs from adult-NAFLD not only in the histological picture but also in the natural history of the disease^28^ might also explain the observed heterogeneity.

Second, raw data of each study was not fully available; that explain why the results (rs58542926-effects) presented in our meta-analysis slightly differ from those in the original studies. In fact, we had to convert some values of medians and interquartile range or range into mean and SD in some of the studies. Third, we could not provide an estimation of the effect of the variant in the additive model because of the low frequency of the T-allele (167 K); thus, all the calculations were based on the dominant model of inheritance. Four, there were differences in the study-design between the two main liver phenotypes; while studies of patients with NAFLD were either population or hospital-based studies that included either control subjects, cases and controls, or cases only; studies of patients with chronic hepatitis were exclusively disease-centric studies that included only cases. Five, we were not able to assess the effect of the variant on the plasma levels of aminotransferases according to sex, because the studies did not disclose data of ALT or AST values separately in men and women. Finally, a limitation that is an intrinsic defect of the cross-sectional design of all the included studies, and which cannot be solved by any specific analysis, is that the values of liver enzymes incorporated into our meta-analysis correspond to the circulating measurement of aminotransferases at only one point in time. Hence, biological oscillations of transaminases, even the ones experimented during the day, after a meal, or exercise^29^, and more importantly, oscillations explained by potential changes in the disease behavior, such as *flare-ups* during the course of chronic hepatitis, could all affect the overall effect, although in a large dataset this noise may be compensated.

### Implications for the understanding of the role of aminotransferases in liver disease

The significant association of rs58542926 and circulating levels of aminotransferases in patients with NAFLD was observed after collecting data from a large dataset consisting of 94,414 individuals for ALT and 93,809 individuals for AST. More specifically, the results of the pooled estimates showed that carriers of the minor T allele (EK + KK individuals) compared with homozygous subjects for the ancestral C allele (EE genotype) have higher levels of liver enzymes. However, it is difficult to attribute a meaningful clinical value to this finding that represents approximately an increase of ~2.5 (9.8%) IU/L of ALT and 1.2 (5%) IU/L of AST, or at least, this finding lacks of a satisfactory clinical interpretation at the population level. For instance, as mentioned earlier, compared with the large effect of the *PNPLA3* rs738409-G allele that is associated with a 28% increase in serum ALT levels^11^, it is hard to attribute the *TM6SF2* locus a direct role in the modulation of circulating levels of transaminases as surrogate indicators of liver damage and/or inflammation. This assumption is partially supported by the divergent results of the association of rs58542926 and aminotransferases in NAFLD vs. chronic hepatitis. Notably, compared with NAFLD, though in a smaller but adequate statistical power- sample encompassing 4187 patients with chronic viral hepatitis, the rs58542926 had no effect on the circulating levels of ALT and AST.

What could be the reasons for this apparent discrepancy between the effect of the E167K variant in NAFLD and chronic viral hepatitis?

In patients with chronic hepatitis, rather than liver steatosis, liver damage, inflammation, and necrosis are part of the main histological picture; then, plasma levels of aminotransferases probably reflect liver injury more accurately than metabolic perturbations. An interesting aspect to highlight that reinforces the previous assumption is the significant difference (*p* = 0.0001) between the mean ALT value in the studies of patients with NAFLD (25.3 ± 19.5 IU/L) compared that of the studies of patients with chronic hepatitis (72.49 ± 167.82 IU/L). Similarly, the plasma levels of AST significantly (*p* = 0.0001) differ between the studies of patients with NAFLD (23.96 ± 15.3 IU/L) versus studies of patients with chronic hepatitis (51.98 ± 124.91 IU/L). These differences probably reflect a distinctive clinical meaning of the elevated levels of these enzymes in each of the two phenotypes. For instance, in patients with chronic viral hepatitis, the elevation of aminotransferases in the order of ~ two times or more the upper normal limit most likely represents histological changes associated with hepatocellular injury. On the contrary, circulating ALT or AST values in NAFLD, which are hardly ever in the range of chronic hepatitis and can be even in the “normal” range, do not necessarily represent histological severity but rather metabolic perturbations^4^,^30^,^31^. Several observations support the notion that aminotransferases are not only induced in NAFLD^4^,^32^, but that the liver gene expression of transaminase isoforms correlates with the ALT and AST levels in the circulating compartment, along with metabolites of the Krebs cycle^4^. For instance, long-term follow-up results from the Framingham Offspring Heart Study suggest that aminotransferases are associated with the long-term development of multiple metabolic disorders^3^. In addition, results from large epidemiological studies worldwide, such as the National Health and Nutrition Examination Survey (NHANES) have consistently demonstrated that elevations of aminotransferases in the general population are associated with risk factors for NAFLD, including central adiposity and hyperinsulinemia^33^,^34^. Similar studies from Asia (National Health and Nutrition Examination Survey K-NHANES) replicated these results not only in the adult population^35^, but also in the adolescent population^36^.

Therefore, based on all of the above-mentioned evidence, we are prompted to formulate the hypothesis that the E167K variant is associated with circulating levels of aminotransferases as an indirect consequence of liver metabolic perturbations in the context of MetS and NAFLD, but not necessarily with liver injury or necrosis. Consequently, in population-based studies on NAFLD or MetS that do not include patients with liver biopsy, associations between the E167K variant and plasma levels of aminotransferases should not be regarded as surrogates of liver damage, but rather a derived-phenotype associated with liver fat overload.

The accumulation of supporting experimental evidence on the functional role of the E167K variant and TM6SF2 gene and protein strengthen our hypothesis. For instance, previous studies on the biological function of *TM6SF2*, either *in vitro*^37^ or *in vivo* by knockdown of *Tm6sf2* in mice^12^,^38^, consistently demonstrated a critical role in lipoprotein metabolism, specifically in the secretion of very-low-density lipoproteins. The allelic-specific expression analysis of cDNA isolated from the liver tissue of patients with NAFLD confirmed that the expression levels of rs58542926-T allele are about 56% of that of the C allele^24^; also, patients with NAFLD have a reduced TM6SF2 protein expression in the liver^24^. Remarkably, a recent experimental work that used novel, genetically engineered, transgenic mouse models, such as mice that express TM6SF2 in the liver specifically, and also mice with CRISPR/Cas9-mediated knockout of *Tm6sf2*, showed that *TM6SF2* did not alter either ALT or AST levels^38^. In addition, feeding with a high fat diet for 10–12 weeks did not induce either inflammation or the development of significant liver fibrosis^38^; furthermore, liver expression of tumor necrosis factor α and monocyte chemoattractant protein-1 were neither induced in *TM6SF2* KO mice^38^.

Finally, the current evidence on human studies suggest that the E167K variant exerts a modest effect on liver fat accumulation, as the carriers of the K-risk allele have a ~2.13-fold higher risk of developing NAFLD, and also show an approximately ~2.2–4% (depending on the inheritance-model) higher hepatic fat content when compared with carriers of the EE genotype^14^. It is reasonable to then speculate that this modest effect on the risk of NAFLD is consequently associated with a modest effect on the disease severity, and specifically on liver damage and inflammation; unfortunately, a large proportion of studies included in this meta-analysis lack genotypes counts according to liver biopsy inflammatory scores, thereby precluding any further analysis. By contrast, the G-risk allele of *PNPLA3*-rs738409 that is associated with a 3.26-fold risk of developing NAFLD^11^ is associated with a 3.24-fold higher risk of higher necroinflammatory scores when compared with homozygous subjects for the C allele^11^. In conclusion, it is plausible to suggest that the association of rs58542926 with aminotransferase levels might be regarded as an epiphenomenon of the liver metabolic perturbations observed in NAFLD rather than a direct influence of the variant on liver damage or inflammation.

## Material and Methods

### Data Sources and Study Selection

Electronic searches of Pubmed at the National Library of Medicine (http://ncbi.nlm.nih.gov/entrez/query), Google Scholar and EMBASE and the Science Citation Index databases were performed using the search terms “TM6SF2” in all fields, and “rs58542926, gene or variants or polymorphism or alleles”. In addition, citations in retrieved articles as well as articles disclosed by the PubMed “related articles” link were further evaluated for inclusiveness. All the published evidence until March 2016 without any country restriction was included in the initial assessment; 48 studies were selected for the analysis.

The search and the assessment of the eligibility criteria was conducted by the authors (SS and CJP), who independently performed this task; there were no discrepancies in this process and the inter-observer variability by calculating the kappa statistic was 1.0. A detailed description of the process of data collection, including identification, screening, eligibility and selection of the studies can be found in the Supplementary Fig. 1.

This meta-analysis followed the appropriate methods for conducting the meta-analysis of genetic association studies, as stipulated in The Human Genome Epidemiology Network (HuGENet) guidelines (http://www.cdc.gov/genomics/hugenet/participate.htm), which are specific for the assessment of the strength of evidence for gene-phenotype associations.

### Inclusion and Exclusion Criteria for Data Source Selection

The protocol that includes the eligibility criteria for selecting studies in our meta-analysis is as follows: candidate gene association studies, either population-based or hospital-based case-control, and GWAS concerning the *TM6SF2* rs58542926, in which information on ALT and AST values as well as the number of subjects in each genotype was given, sufficient data to calculate outcomes was available, and genotyping was performed using a validated molecular method. As the literature search showed reports of the rs58542926 variant in NAFLD and viral hepatitis, a separate analysis for each main phenotype was conducted.

The exclusion criteria were as follows: duplicate publications, redundant information on genotyped subjects included in more than one study, and unpublished papers. Because the number of homozygous subjects for the T allele is either null in candidate association studies or small in the larger GWAS, we decided to compare the homozygous for the C (Glu167) allele (EE genotype) vs. the carriers of the T allele (Lys167), specifically the heterozygous EK+ homozygous KK genotypes, as explained earlier^14^. For each phenotype, we evaluated the association results stratified by age and ethnicity.

### Data Collection

From each study included in our meta-analysis, we retrieved relevant information on demographics, such as age, sex, and ethnicity, and circulating levels of ALT and AST expressed in international units (IU/L) and measured by any standard analytical method (variables expressed as mean ± standard deviation, SD); standard error and interquartile rank were converted to SD. Data on the explored phenotypes was extracted for the EE and EK+KK genotypes, and the analyses were based on comparing the genotype groups without any further adjustment for confounding factors.

### Statistical Analysis

While the main outcome (ALT and AST levels) was measured in IU/L across all studies, the expression of data was not always uniform, and potential variability across different laboratories could also be expected. Then, we attempted to homogenize the results through the use of standardized mean difference (D), which is the difference in means pertaining to cases and controls divided by the common within-group SD. The difference in means was used to estimate the magnitude of the absolute effect in IU/L. The statistical protocol applied to this study was already published in our previous work on the meta-analysis of the role of rs58542926 on lipid traits and NAFLD^14^. While both the fixed and random effect models were assessed for all the explored variables, the random model was used to summarize statistical synthesis, as it assumes that the treatment effect is not the same across all of the studies. Thus, the goal was to estimate the average effect in the studies^39^, assuming that ethnicity or age may provide an important source of variability. Heterogeneity was evaluated with the Q statistic and *I*^2^ statistic, which is a transformation of Q that estimates the proportion of the variation in effect sizes that is due to heterogeneity between the studies. In such cases, an *I*^2^ value of 0% indicated no observed heterogeneity, while greater values corresponded to increasing heterogeneity. In the case of heterogeneity, we identified study characteristics that stratified the studies into subsets with homogeneous effects. We considered the possible sources of heterogeneity and stratified the studies by age and ethnicity, and also repeated the analysis separately for each group. If the association became homogeneous after stratification or after excluding the outlier studies, we recalculated the overall effect and 95% CI, and no further action was taken. Although the studies excluded in this way cannot be considered outliers, excluding studies that contribute the most to heterogeneity is an unbiased way of achieving the homogeneity required for a stringent meta-analysis^39^. To check for publication bias, we used a visual inspection of funnel plots and Begg and Mazumdar’s rank correlation test (this test, which is also known as rank correlation coefficient or simply Kendall’s τau, reports the rank correlation between the standardized effect size and the variances, or standard errors, of these effects)^40^. A *p* value of ≤0.05 was considered statistically significant. All calculations were performed using the Comprehensive Meta-Analysis computer program (Biostat, Englewood, NJ, USA).

## Additional Information

**How to cite this article**: Sookoian, S. and Pirola, C. J. Meta-analysis of the influence of *TM6SF2* E167K variant on Plasma Concentration of Aminotransferases across different Populations and Diverse Liver Phenotypes. *Sci. Rep.* **6**, 27718; doi: 10.1038/srep27718 (2016).

## Supplementary Material

Supplementary Information

## Figures and Tables

**Table 1:** The association between the nonsynonymous rs58542926 variant of *TM6SF2* and aminotransferase (ALT and AST) levels in studies of patients with nonalcoholic fatty liver disease (NAFLD): Characteristics of the studies and potential bias.

		Main clinical descriptors of the studies	Genotyping approach and HWE*	Comments on potential concerns and bias	ALT mean ± SD		AST mean ± SD	
Study ref. and Population Ethnicity (*Country*)					EE	EK + KK	EE	EK + KK
**1**	Dongiovanni, 2015^19^ European descendent (*Italy*)	Patients with NAFLD diagnosed by liver biopsy. Age: 42 ± 6 Female %: 48	TaqMan assay. Genotypes were tested and found to be in HWE.	Population stratification untested by the authors. A control group is not available; patients with NAFLD might be recruited due to- but not restricted to- abnormal aminotransferase levels.	44.0 ± 34.1	46.0 ± 32.6	30.0 ± 17.0	30.0 ± 16.3
	Dongiovanni, 2015, b^19^ European descendent (*Finland and Sweden*)	SOS Study: Morbid obese subjects enrolled for bariatric surgery. Age: 49 ± 7 Female %: 70	TaqMan assay. Genotypes were tested and found to be in HWE.	Population stratification untested by the authors.	46.0 ± 20.7	51.0 ± 28.2	35.0 ± 11.9	37.0 ± 14.8
**2**	Eslam, 2016, a^16^ (*European Population*)	Patients with NAFLD diagnosed by liver biopsy. Age: 49 (18–79) Female %: 44	Sequenom MassARRAY system, iPLEX Gold chemistry. Genotypes were tested and found to be in HWE.	Population stratification untested by the authors. A control group is not available, patients with NAFLD might be recruited due to- but not restricted to- abnormal aminotransferase levels.	58.0 ± 115.5	56.7 ± 46.9	40.0 ± 63.3	36.8 ± 31.0
**3**	Grandone, 2015^21^ European descendent (*Italy*)	Obese children >95th percentile recruited to explore the association between lipid traits and hepatic steatosis. Age: 10.5 ± 2.9. Female %: 50	TaqMan assay. Genotypes were tested and found to be in HWE.	Population stratification untested by the authors. A control group is not available; data is only on obese patients.	28.0 ± 16.1	34.0 ± 18.6	24.2 ± 9.4	25.7 ± 10.5
**4**	Goffredo, 2016, a^20^ Caucasians (*USA*)	Obese children and adolescents recruited from the Yale Pediatric Obesity Clinic. Age: 13 ± 3.5 Female %: 60	Automated sequencing. Genotypes were tested and filtered to be in HWE.	Population stratification untested by the authors. A control group is not available, and data is only on obese patients.	26.3 ± 24.1	36.0 ± 59.0	24.0 ± 11.6	29.4 ± 36.0
	Goffredo, 2016, b^20^ African Americans (*USA*)	Age: 13.2 ± 3 Female %: 60	Automated sequencing. Genotypes were tested and filtered to be in HWE.	Population stratification untested by the authors. A control group is not available, and data is only on obese patients.	16.9 ± 8.6	14.2 ± 5.7	21.4 ± 6.3	20.8 ± 5.7
	Goffredo, 2016, c^20^ Hispanics (*USA*)	Age: 13 ± 3 Female %: 50	Automated sequencing. Genotypes were tested and filtered to be in HWE.	Population stratification untested by the authors. A control group is not available, and data is only on obese patients.	26.4 ± 21.5	47 ± 60	24.7 ± 9.3	38.5 ± 42
**5**	Kozlitina, 2014, a^12^ Mixed population: Hispanics/African-Americans/European-Americans (*USA*)	Dallas Heart Study: individuals recruited for exploration of CV risk factors and fatty liver. Age: 46 ± 11 Female %: 57	Illumina Infinium HumanExome BeadChip. Genotypes were tested and filtered to be in HWE.	Population stratification was computed by ancestry markers.	23.5 ± 19.9	25.9 ± 18.3	24.3 ± 20.6	25.1 ± 19.5
	Kozlitina, 2014, b^12^ European-Americans (*USA*)	Preventive medicine, Dallas Biobank Texas Age:53±11 Female %:35	Taqman assay. Genotypes were tested and found to be in HWE.	Population stratification unlikely: 100% participants European-Americans	35.9 ± 16.3	36.9 ± 15.5	27.0 ± 13.8	27.1 ± 10.9
	Kozlitina, 2014, c^12^ 12 Danish population (*Sweden*)	Copenhagen General Population Study and the Copenhagen City Heart Study. Age: 58 ±10 Female %: 55	Taqman assay Genotypes were tested and found to be in HWE.	Population stratification unlikely: all participants were of Danish descent.	22.7 ± 17.2	23.9 ± 16.3	22.8 ± 13.2	24.2 ± 19.8
**6**	Krawczyk 2016^22^ Polish Caucasian (*Poland*)	Hospital-based Enrolled to assess dietary intervention Age: 48 (18–74) Female %: 38	Taqman assay Genotypes were tested and found to be in HWE.	Population stratification untested by the authors.	46 ± 66.25	42.7 ± 31.3	ND	ND
**7**	Scorletti E^23^2015 European descendent (*England*)	Patients recruited in a controlled trial on the effect of 4 g per day of a omega-3 fatty acid. Age: 50 ± 9.6 Female %: 17	TaqMan assay. HWE not tested; calculations cannot be done because the authors did not disclose full genotypes counts.	Population stratification untested by the authors.	55.0 ± 51.0	55.0 ± 58.0	42.0 ± 24.0	39.0±40.0
**8**	Sookoian, 2015^24^ European descendent Caucasian (*Argentina*)	Case-control study; patients with NAFLD diagnosed by liver biopsy. Age: 50 ± 12 Female %: 62	TaqMan assay. Genotypes were tested and found to be in HWE.	Population stratification tested and negative.	55.5 ± 52.2	68.5 ± 82	40.07 ± 29	40.4 ± 26.3
**9**	Wong, 2014^26^ Chinese (*China*)	Subjects assessed for non-invasive screening of fatty liver and liver fibrosis. Age: 46 ± 10 Female %: 58	TaqMan assay. Authors did not report HWE testing, but our calculations show that all genotype frequencies were in HWE	Population stratification untested but unlikely as the subjects were restricted to Chinese.	21.0 ± 10.4	22.2 ± 9.1	19.0 ± 4.44	20.1 ± 8.2
**10**	Zhou, 2015^27^ Finnish (*Finland*)	Subjects recruited for performing metabolic studies; target population obese and T2D patients. Age: 48 ± 1.5 Female %: 38	Allele-specific fluorescence. Genotypes were tested and found to be in HWE.	Population stratification untested by the authors.	34.0 ± 23.7	34.0 ± 17.8	29 ± 15.6	30.0 ± 9.6
**11**	Viitasalo, 2016^25^ Finnish (*Finland*)	Cross-sectional population-based study to assess physical activity and diet intervention study Age: 7.6 ± 0.4 Female %: 49	Exome BeadChip Genotypes were tested and found to be in HWE.	Population stratification untested by the authors.	21.0 ± 8.5	18.3 ± 5.8	ND	ND

HWE: Hardy-Weinberg equilibrium. Age expressed in years, mean ± standard deviation or mean (range) as stated in the referenced manuscripts. CV: cardiovascular. ND: Not done.

**Table 2:** The association between the nonsynonymous rs58542926 variant of *TM6SF2* and aminotransferase (ALT and AST) levels in studies of patients with chronic viral hepatitis C (HCV) or B (HBV): Characteristics of the studies and potential bias.

		Main clinical descriptors of the studies	Genotyping approach and HWE*	Comments on potential concerns and bias	ALT mean ± SD		AST mean ± SD	
Study ref. and Population Ethnicity (*Country*)					EE	EK + KK	EE	EK + KK
**1**	Eslam, 2016, b^16^ European Population	HCV Age: 44.8 ± 10.7 Female %: 37.7	Sequenom MassARRAY system and iPLEX Gold chemistry. Genotypes were tested and found to be in HWE.	Population stratification untested by the authors.	76.95 ± 226.0	85.2 ± 163.9	53 ± 120.5	58.9 ± 72.3
	Eslam, 2016, c^16^ Chinese Population	HBV Age: 43.3 ± 11.6 Female %: 32.7	Sequenom MassARRAY system and iPLEX Gold chemistry. Genotypes were tested and found to be in HWE.	Population stratification untested by the authors.	63.0 ± 173.5	65.0 ± 117.3	44.0 ± 179.0	52.0 ± 41.0
**2**	Coppola, 2015^15^ European descendent (*Italy*)	HCV Age 52 ± 12.9 Female %: 45	TaqMan assay. HWE not tested; calculations cannot be done because the authors did not disclose full genotypes counts.	Population stratification untested by the authors.	79.0 ± 61.0	88.6 ± 70.0	52.0 ± 35.0	57.0 ± 34.0
**3**	Petta S, 2015^18^ European descendent (*Italy*)	HCV Age: 53 ± 11 Female %: 40	TaqMan assay. Genotypes were tested and found to be in HWE.	Population stratification untested by the authors.	88.1 ± 73.8	76.5 ± 51.2	ND	ND
**4**	Milano, 2015^17^ European descendent (*Italy*)	HCV Age: 57.5 ± 13 Female %: 39.5	TaqMan assay. Genotypes were tested and found to be in HWE.	Population stratification untested by the authors.	51.0 ± 49.6	47.0 ± 43.7	ND	ND

HWE: Hardy-Weinberg equilibrium. ND: not done. Age expressed in years, mean ± standard deviation or mean (range) as stated in the referenced manuscripts.
