# Supplementary material for: Meta-analysis of the influence of TM6SF2 E167K variant on Plasma Concentration of Aminotransferases across different Populations and Diverse Liver Phenotypes
Source: Sci Rep. 2016 Jun 9;6:27718. doi: 10.1038/srep27718 (PMC4899730; doi:10.1038/srep27718)

**SUPPLEMENTARY INFORMATION**

**Meta-analysis of the influence of *TM6SF2* E167K variant on Plasma Concentration of Aminotransferases across different Populations and Diverse Liver Phenotypes**

Silvia Sookoian^1^ MD, PhD. and Carlos J Pirola^2^ PhD, FAHA, FASHG

^1^Department of Clinical and Molecular Hepatology, Institute of Medical Research A Lanari-IDIM, University of Buenos Aires- National Scientific and Technical Research Council (CONICET), Ciudad Autónoma de Buenos Aires, Argentina

^2^Department of Molecular Genetics and Biology of Complex Diseases, Institute of Medical Research A Lanari-IDIM, University of Buenos Aires-National Scientific and Technical Research Council (CONICET), Ciudad Autónoma de Buenos Aires, Argentina

**Supplementary figures**

**Supplementary Figure 1**

#### **Search strategy, i**nclusion and exclusion criteria.

**
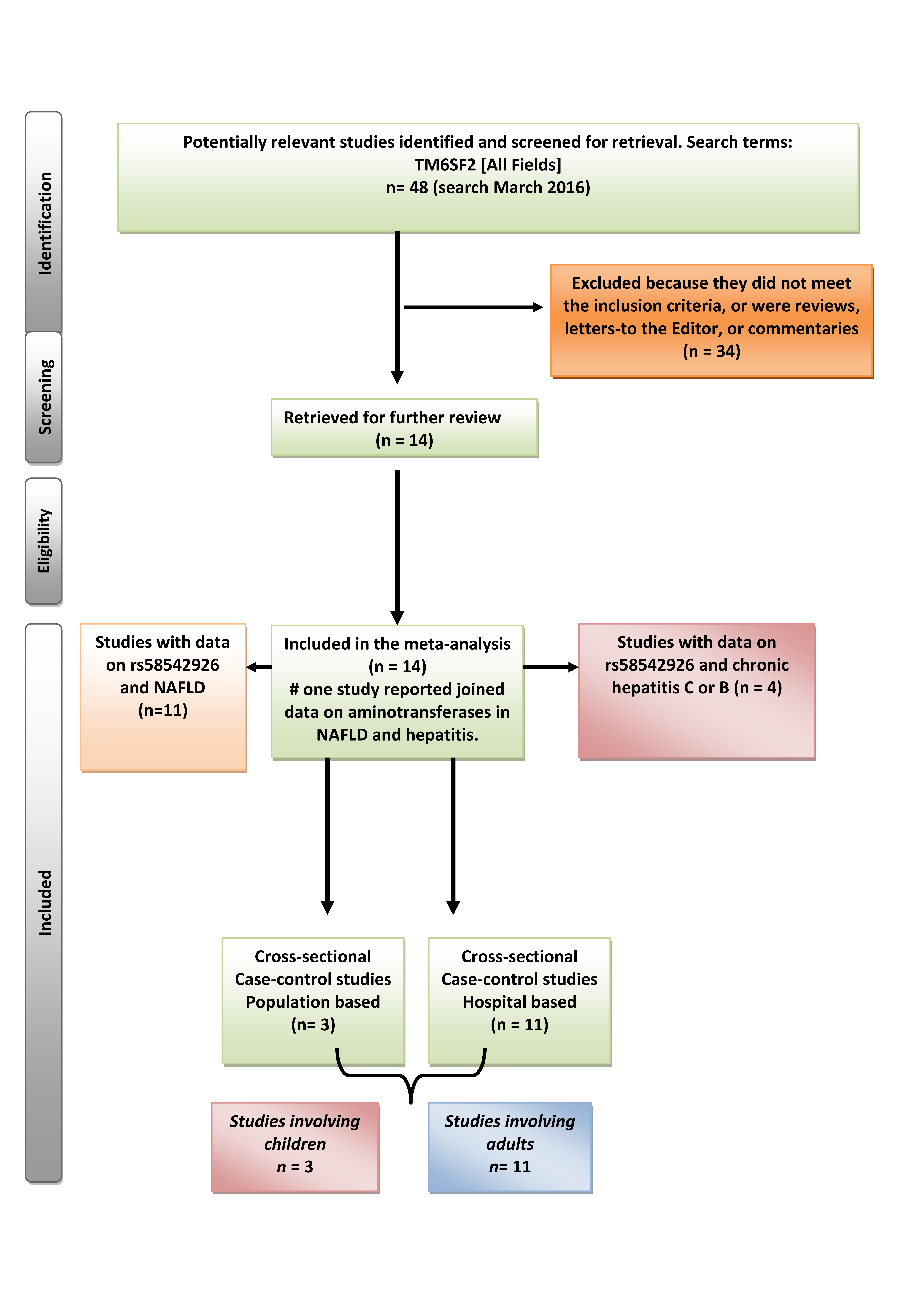
**

**Supplementary Figure 2**

Forest plot of rs58542926 variant (homozygous EE vs. EK+KK) and plasma level of ALT in patients with NAFLD stratified by age. The effect indicates the standardized mean difference, the standard error, and the corresponding lower and upper limits. Studies were ordered by sample size.


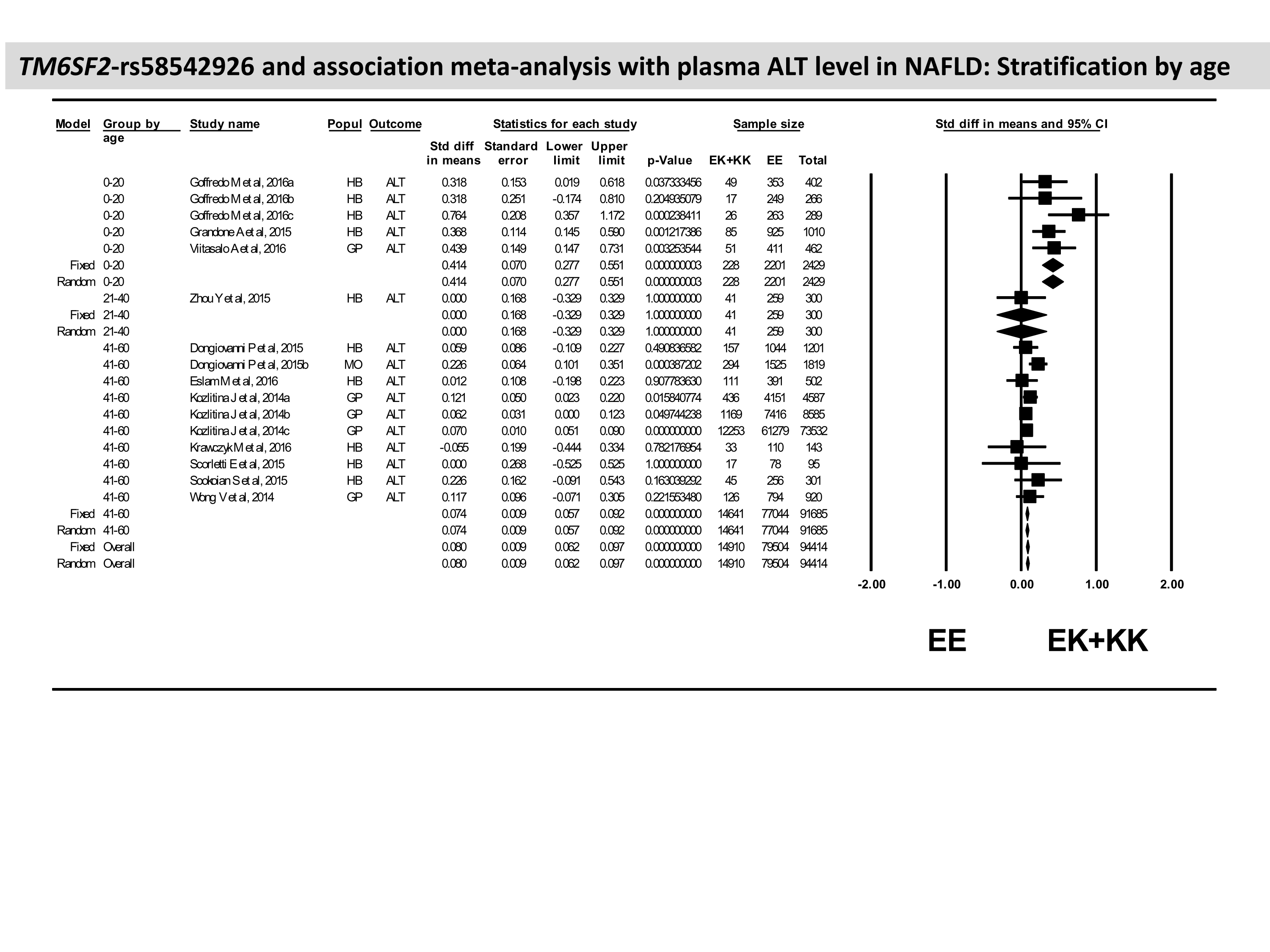


**Supplementary Figure 3**

Forest plot of rs58542926 variant (homozygous EE vs. EK+KK) and plasma level of AST in patients with NAFLD stratified by age. The effect indicates the standardized mean difference, the standard error, and the corresponding lower and upper limits. Studies were ordered by sample size.


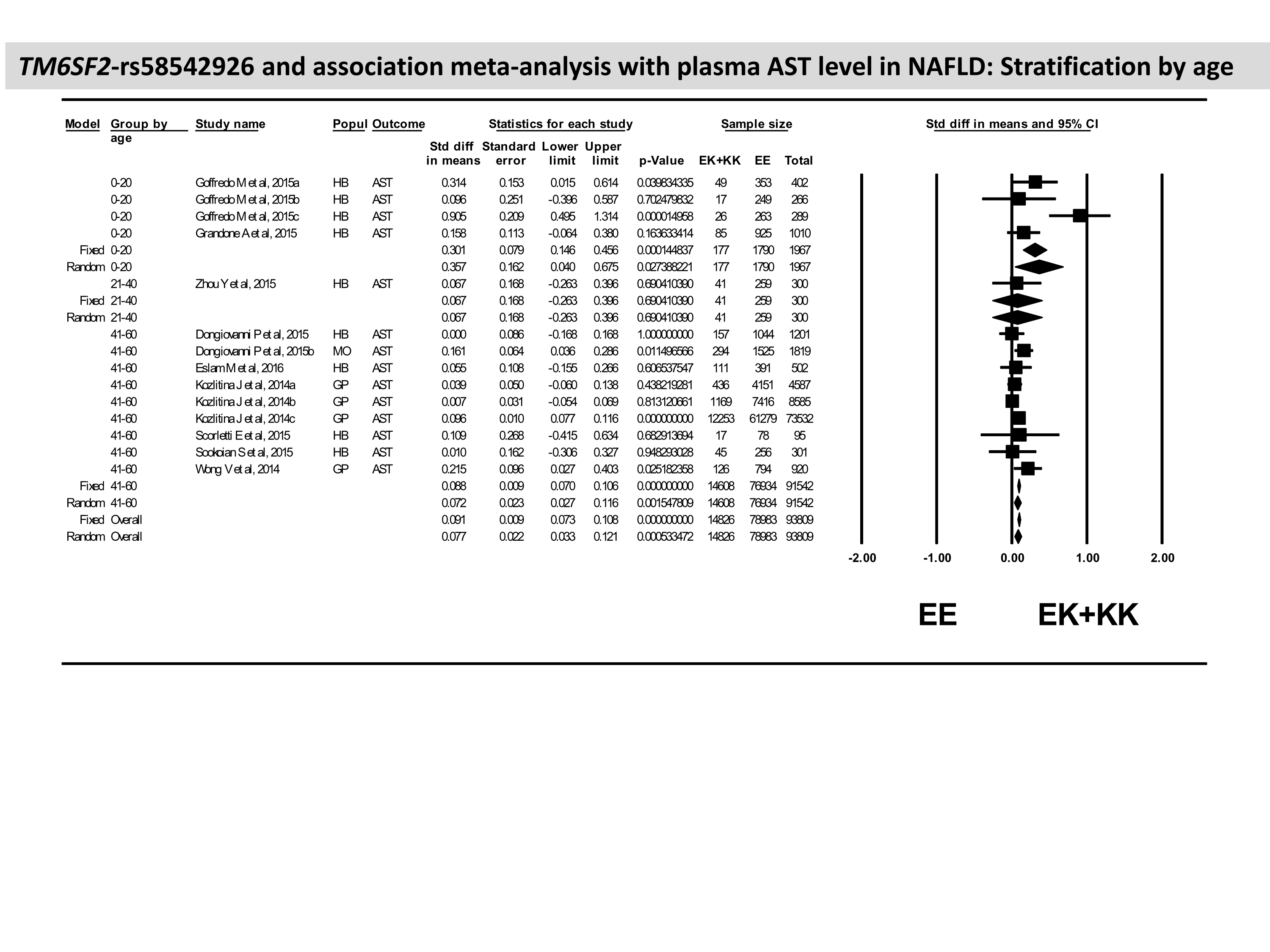

Supplement: Supplementary Information [file srep27718-s1.doc]
